# Supplementary material for: Staphylococcus aureus α-Toxin Effect on Acinetobacter baumannii Behavior
Source: Biology (Basel). 2022 Apr 9;11(4):570. doi: 10.3390/biology11040570 (PMC9028598; doi:10.3390/biology11040570)
Supplement: Supplementary file 1 [file biology-11-00570-s001.zip › Table S1.pdf]

Table S1: Tetracycline and Imipenem MICs of A118, A42, AB5075 grew in LB or LB + 50% CFCM

Conditions	MIC TET (µg/mL)	MIC IMI (µg/mL)
A118 LB	14 ± 2 (I)	24 ± 0 (R)
A118 $CFCM_{USA300}$	14 ± 2 (I)	23 ± 2 (R)
A118 $CFCM_{USA300\ \Delta \mathit{hla}}$	14 ± 3 (I)	25 ± 2 (R)
A118 $CFCM_{USA300\ \Delta \mathit{hla}\ comp}$	14 ± 2 (I)	24 ± 0 (R)
A42 LB	12 ± 2 (I)	24 ± 2 (R)
A42 $CFCM_{USA300}$	20 ± 2 (R)	25 ± 3 (R)
A42 $CFCM_{USA300\ \Delta \mathit{hla}}$	14 ± 2 (I)	23 ± 2 (R)
A42 $CFCM_{USA300\ \Delta \mathit{hla}\ comp}$	15 ± 3 (I)	24 ± 0 (R)
AB5075 LB	4 ± 2 (S)	12 ± 2 (R)
AB5075 $CFCM_{USA300}$	14 ± 2 (I)	12 ± 2 (R)
AB5075 $CFCM_{USA300\ \Delta \mathit{hla}}$	3 ± 2 (S)	4 ± 0 (I)
AB5075 $CFCM_{USA300\ \Delta \mathit{hla}\ comp}$	10 ± 2 (I)	5 ± 0 (R)

S: Susceptible, I: Intermediate, R: Resistant
