# Supplementary figures and images for: Staphylococcus aureus α-Toxin Effect on Acinetobacter baumannii Behavior
Source: Biology (Basel). 2022 Apr 9;11(4):570. doi: 10.3390/biology11040570 (PMC9028598; doi:10.3390/biology11040570)

## ZOC (t 120 min)

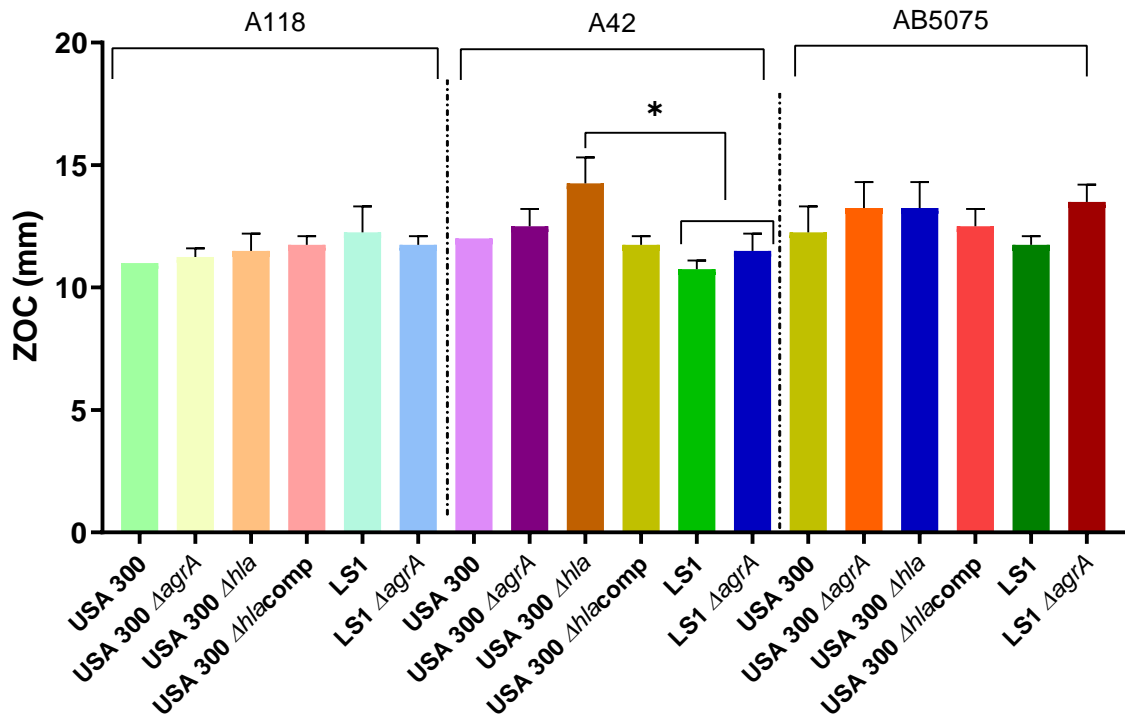

Supplement: Supplementary file 1 [file biology-11-00570-s001.zip › Figure S1.pdf]
